# Cocreation of a Video Feedback Tool for Managing Self-Care at Home With Pairs of Older Adults: Remote Experience-Based Co-Design Study

**DOI:** 10.2196/57219

**Published:** 2024-10-28

**Authors:** Susanna Strandberg, Mirjam Ekstedt, Cecilia Fagerström, Sofia Backåberg

**Affiliations:** 1 Department of Health and Caring Sciences Linnaeus University Kalmar/Växjö Sweden; 2 Department of Learning, Informatics, Management and Ethics Karolinska Institutet Stockholm Sweden; 3 Department of Research Region Kalmar County Kalmar Sweden; 4 Faculty of Kinesiology University of Calgary Calgary, AB Canada

**Keywords:** chronic illness, eHealth, experience-based co-design, older adults, self-care, video feedback

## Abstract

**Background:**

Involving older adults in co-design processes is essential in developing digital technologies and health care solutions to enhance self-care management at home, especially for older adults with chronic illness and their companions. Remote co-design approaches could provide technologically sustainable solutions that address their personal needs.

**Objective:**

This study aimed to cocreate and test the usability of a video feedback tool to facilitate self-care management at home.

**Methods:**

This experience-based co-design approach involved collaboration between 4 pairs of older adults, 4 researchers, and 2 service designers in three steps: (1) six iterative workshops (5 remote and 1 in person) to cocreate self-care exercises within an existing video feedback tool by identifying factors influencing self-care management; (2) developing and refining the self-care exercises based on suggestions from the older adults; and (3) usability testing of the cocreated exercises with the 4 pairs of older adults in their homes. Among the older adults (68-78 years), 3 adults had heart failure and 1 adult had hypertension. Data were analyzed inductively through thematic analysis and deductively using the USABILITY (Use of Technology to Engage in Adaptation by Older Adults and/or Those With Low or Limited Literacy) framework.

**Results:**

The identified influencing factors guiding the contents and format development of 2 new self-care exercises were that pairs of older adults support and learn from each other in performing self-care, which increases their motivation and engagement in practicing self-care at home. The usability test of the 2 new self-care exercises, “Breathing exercises” and “Picking up from the floor,” revealed that the pairs found the exercises and the video feedback component valuable for learning and understanding, for example, by comparison of performances highlighting movement variability. However, they found it difficult to manage the video feedback tool on their own, and a support structure or tailored education or training was requested.

**Conclusions:**

This study emphasizes that the video feedback tool holds the potential to facilitate learning and understanding in self-care management, which may support motivation. The studied video feedback tool can be beneficial for pairs of older adults managing self-care at home as a complement to traditional health care services, but an accurate supporting structure is required. The effectiveness of the video feedback tool and its integration into existing health care services still need to be assessed and improved through careful design and structured support.

## Introduction

eHealth, an evolving field at the intersection of medical informatics, health care, and business, actively advances global health care through enhanced service delivery and information dissemination via the internet and related technologies [1]. As eHealth technologies and remote care become integral to everyday health care services, facilitating self-care management [2-4], it is necessary to involve older adults in co-design processes [5,6]. Older adults’ engagement can ensure that these digital tools meet their specific needs, promoting user acceptance and enhancing usability, accessibility, and effectiveness [5,6]. However, many digital tools used by older adults at home have both advantages and disadvantages [7-9], particularly for those with chronic illnesses. A lack of adaptation to user contexts and interactions [10] highlights the need for better-designed solutions for self-care management [3,11].

Performing self-care is crucial for living an independent life in old age, including when managing the challenges that come with natural aging [12]. The importance of developing effective digital tools tailored to older adults’ needs is underscored by the rising prevalence of chronic illnesses such as hypertension and heart failure. This rise is linked to the increasing proportion of older adults worldwide [13,14]. Elevated blood pressure and age increase the risk of cardiovascular diseases like heart failure. Alongside medication, self-care management and lifestyle changes related to diet, weight loss, and physical activity effectively mitigate this risk for older adults [15].

Self-care, defined as maintaining health through health-promoting practices and managing illness, includes daily activities related to personal hygiene, diet, and mobility exercises. Self-care involves promoting the ability and motivation to perform self-care exercises consistently over time, making it essential in both healthy and ill states [16,17]. Old age, especially when accompanied by chronic illness, can lead to dependency on daily activities due to aging-related issues such as disability, mobility problems, cognitive decline, falls, malnutrition, and communication difficulties. These issues negatively impact the quality of life, increasing the importance of self-care interventions [18,19].

Collaborative self-care efforts between individuals, particularly when functioning as a pair, have shown notable benefits and mutual encouragement over time [20]. Social support is important for self-care management at home and could support sustainable health and reduce the burden on the health care system [21]. Recognizing the crucial role of companions in facilitating self-care for older adults with chronic illness is essential for reducing the burden on ill adults and enhancing self-care outcomes [22]. Furthermore, the integration of digital tools and peer support could enhance healthier behavior and confidence in self-care management among older adults with chronic illness [20,23,24]. Involving older adults in cocreation is essential to creating tailored health care and self-care support, enabling the development of advanced solutions for effective health care delivery [25,26]. However, there is a remaining need to deepen the understanding of how older adults can benefit from digital tools to achieve sustainable self-care management at home [7,27].

Previous studies among younger adults and children have demonstrated that digital tools incorporating video feedback and reflection have the potential to enhance motivation and facilitate learning by encouraging reflection and active engagement [28,29]. This has been seen in the performance of various sports activities [28-30] and ergonomic skills training among nursing students [31]. Video feedback combined with peer support, open inquiry, and self-reflection—including allowing users to see themselves performing a movement and analyze their performance with open and guiding questions—may improve engagement and motivation by encouraging users to reflect on their actions with their own words. This process may enhance awareness of individual needs and serve as motivation for long-term behavioral changes [31]. In this study, pairs of older adults were engaged in the co-design of a video feedback tool in the context of self-care management at home. Pairs of older adults were involved as social support was considered to have a potential for enhancement of sustainable self-care and to enable the use of video feedback based on peer support. This study aimed to cocreate and test the usability of a video feedback tool to facilitate self-care management at home.

## Methods

### Study Design

The study was based on the Medical Research Council’s framework for developing and evaluating complex interventions [32]. The study adopted a participatory research design in an iterative cyclical process of experience-based co-design (EBCD) to integrate end users’ experiences into service and intervention development. Active involvement of end users during the initial stages allowed them to articulate their preferences and experiences effectively [33,34]. Three co-design teams of end users, researchers, and designers collaborated to identify user needs and develop, refine, and test a video feedback tool [33,35]. The “co” in EBCD signifies a partnership for innovative service or product design, leveraging the co-designers’ expertise in user experience [36].

### A 3-Step Procedure

**Figure 1:**
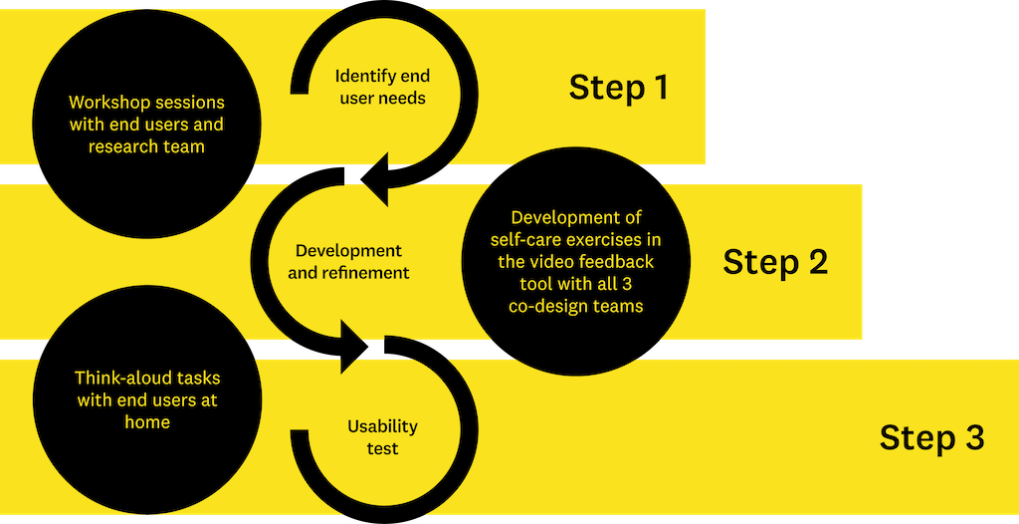
Overview of the 3 study steps of the experience-based co-design process used in the study.

The EBCD process in this study consisted of 3 steps (Figure 1). The first step, identifying end users’ needs, included 6 workshop sessions (5 remote and 1 in person). The research team developed a brief plan and concepts for each workshop, including an introduction to an existing video feedback tool. The strategy and activities were iteratively refined based on the outcomes of each session, enabling an adaptable approach. The second step involved developing and refining self-care exercises within the video feedback tool. Suggestions from the workshops with end users’ preferences were used to cocreate new self-care exercises within the tool, in collaboration with all 3 co-design teams involved. The third step was usability testing of the cocreated self-care exercises from step 2 in the home environment using a think-aloud technique [37].

Each end user pair received an Apple iPad prepared with the video feedback tool and video conferencing software for use during the project period, from March 2021 to March 2022. The video conferencing software facilitated remote workshops. iPads were delivered in person, adhering to COVID-19 guidelines.

### The Video Feedback Tool

**Figure 2:**
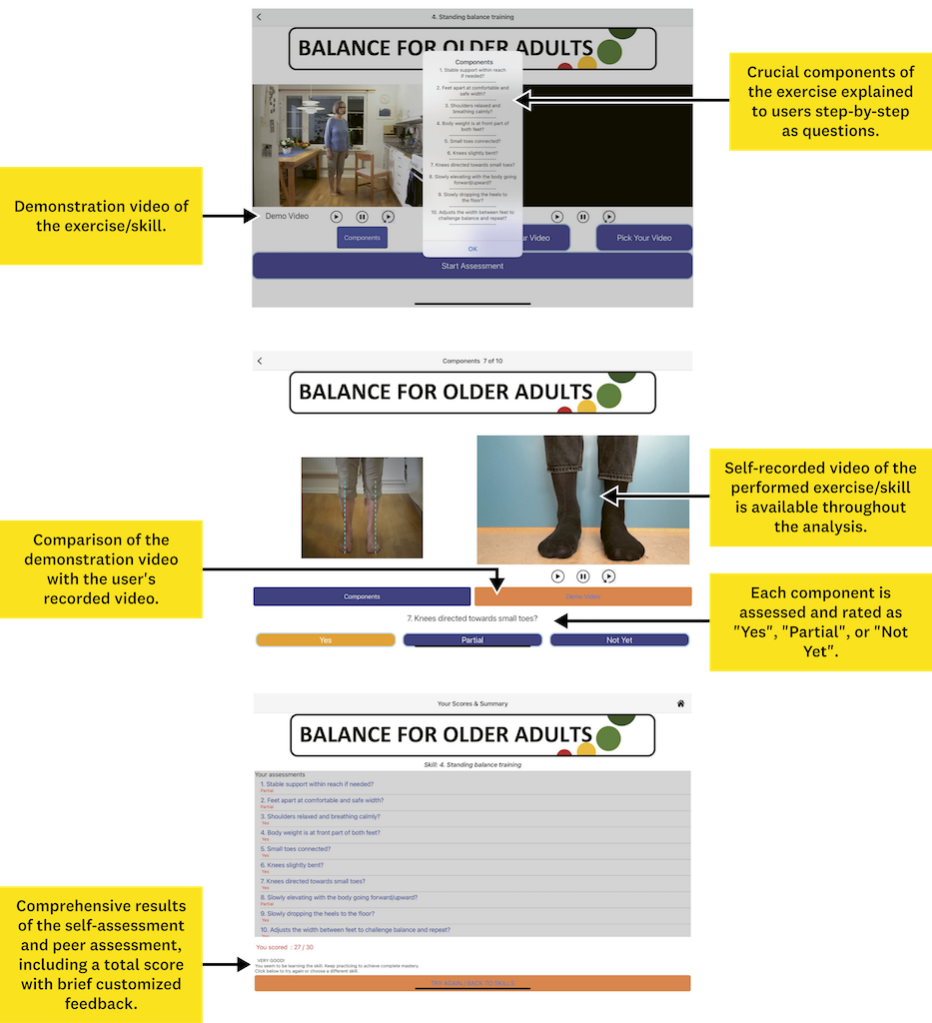
An example of the features within the Move Improve platform, including the demonstration video, the list of components, the self-assessment and peer assessment feature, and the assessment score with personalized written feedback.

For this study, the existing digital platform “Move Improve” was used, as it offers the possibility of facilitating skill acquisition through peer-to-peer learning and video feedback. Move Improve enables users to self-reflect and reflect with peers, based on self-recorded videos, and guided by key component questions, with the overarching goal of mastering skills [38]. Notably, Move Improve provides the opportunity for content creation by developers, and it has previously undergone development and testing in various contexts [29,31]. At the start of the process, the platform included 2 self-care exercises targeting older adults: “Balance for older adults” and “Daily life movements.” These were originally developed in a related research project on physical functioning for older adults. Upon selecting an exercise, users received step-by-step instructions (guiding questions of critical components) for its performance, complemented by a video demonstration and clarifying images for each component (Figure 2). The other person in the pair would record the user while they performed the exercise, facilitating subsequent analysis based on individual components. The analysis involved comparing the user’s performance with the exercise instructions, allowing for ratings of “Yes,” “Partial,” or “Not Yet” (Figure 2). This self and peer assessment resulted in a final score and a written comment provided to the user (Multimedia Appendix 1).

### Participants as Co-Designers

The co-design teams involved the following: the older adults and their companions in pairs, hereafter called “end users”; the research team; and the design team (Table 1)*.* End users—individuals with chronic illness—were purposely recruited by the first author via telephone from a related study testing telemonitoring devices in a primary health care setting in southern Sweden [20]. The inclusion criteria were the ability to read and speak Swedish, being >65 years old, having 1 or more chronic illnesses, and having internet access. Additionally, participants were encouraged to include a companion, forming pairs for this study. The relationship with the companion could vary, but regular contact was essential. Both members of each pair had to reside in private homes, although not necessarily the same home. Initially, 5 persons were invited to participate and asked to bring a companion. One person declined without providing an explanation. Ultimately, 8 older adults participated in the study, forming 4 pairs (Table 1).

**Table 1:** Demographic information of the co-designers involved in all 3 steps of the study.

Co-designers and professions		Professionals, n	Age (years; male)	Age (years; female)	Relationship with companion	Chronic illness
Older adults pair 1^a^		—^b^	—	68 and 68	Friends, not living together	Hypertension
Older adults pair 2		—	77	77	Partners, living together	Heart failure
Older adults pair 3		—	78	77	Partners, living together	Heart failure
Older adults pair 4		—	76	68	Partners, living together	Heart failure
**Research team**						
	Registered nurse	3	—	—	—	—
	Registered physiotherapist	1	—	—	—	—
**Design team**						
	Information and communication technology educator	1	—	—	—	—
	Educational psychologist	1	—	—	—	—

^a^Both are female.

^b^Not applicable.

The research team comprised nurses and a physiotherapist trained in nursing, behavioral science, and physiotherapy, bringing diverse expertise to the development of the self-care exercises. The design team consisted of 2 service designers specializing in information and communication technology and learning environments, who contributed to the content and design of the digital platform (Table 1).

### Data Collection

#### Step 1: Identify End User Needs

**Figure 3:**
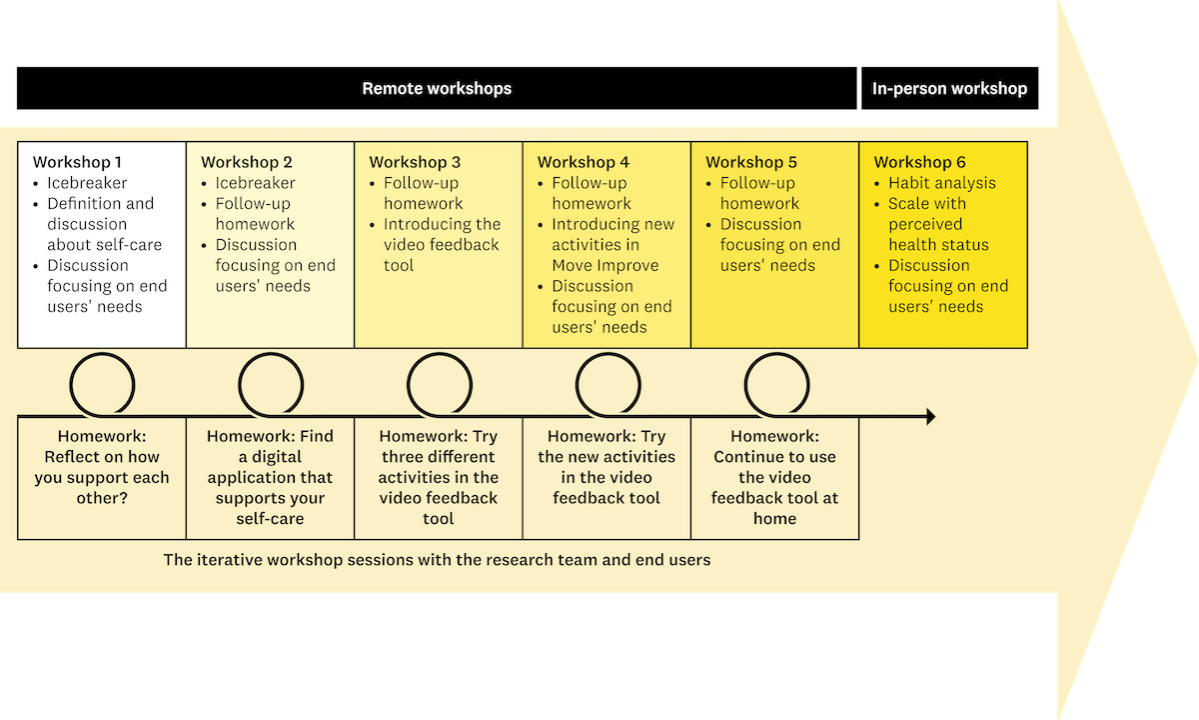
Overview of step 1, the workshop sessions with the research team and the end users, the activities and contents of each workshop, and the homework assigned between sessions.

The research team (SS and SB) and the end user team participated in 6 workshop sessions during Step 1 (Figure 3). The first 2 workshops started with clearly defining the project’s objectives and an icebreaker activity, to promote trust and create a supportive environment [39]. Activities involved presenting a definition of self-care and prompting end users to reflect on their perspectives regarding self-care as well as sharing what contributed to their health and well-being. Each workshop focused on different aspects of the end users’ needs. The video feedback tool, containing 2 exercises created in a related project, was introduced during the third workshop. End users were assigned “homework,” such as reflecting on how they supported each other using the video feedback tool. Figure 3 provides additional details on activities conducted during the workshops. A detailed description and discussion of the methodological and ethical aspects of the workshops and the co-design process were formulated by Backåberg et al [40]. Researchers used a live mind map or digital Post-It notes to engage end users in summarizing each session and wrote memos both during and after each session. Following each session, the research team (SS, SB, and ME) summarized discussions and analyzed their impressions to plan the activities for the next workshop.

During the final in-person workshop, a reflection exercise called “habit analysis” was conducted. End users rated themselves on a scale based on their health status and autonomy [41], aiming to deepen the understanding of their daily experiences and enhance the usability of the cocreated self-care exercises. End users also performed a self-evaluation by placing their names on a scale from less health and more dependence to more health and more independence [41]. End users engaged in brainstorming, scenario creation, rapid prototyping, and refinement of self-care exercises for the video feedback tool. All workshops were audio and video recorded, with researchers observing and taking memos. Each workshop lasted between 60 and 240 minutes, with an average of 125 minutes.

#### Step 2: Development and Refinement

Step 2 involved all 3 co-design teams in the cocreation of the contents of the video feedback tool based on the end users’ experiences shared during workshops (Figure 1). By compiling the end users’ experiences along with the researchers’ Post-It notes and mind maps from the workshop sessions, a list of ideas for new self-care exercises was created. The list of suggested self-care exercises was then discussed with the end users to plan which new self-care exercises would be developed for the video feedback tool. Thereafter, the design team participated in recording videos and taking photographs of these new exercises. In this step, the design team started to make refinements to the Move Improve design based on the feedback received from the end users.

#### Step 3: Usability Test

The usability testing was conducted in the homes of the end users to deliver an authentic end user experience, carried out by 2 researchers (SS and SB). The end users were provided with instructions and tasks by the researchers, that is, to collaboratively select and perform a relevant new self-care exercise from the video feedback tool. One person in the pair was instructed to perform the exercise and recorded the performance with the video feedback tool. Then, they should switch roles and try another exercise. Throughout the usability test, end users were encouraged to verbalize their thoughts and experiences while using the video feedback tool and executing the task, using a think-aloud technique [37] (Figure 1). The usability tests were video recorded and observed by one of the researchers with a structured protocol. The duration of the 8 usability tests was 65-95 minutes with a mean of 75 minutes.

### Data Analysis

All recorded workshops from Step 1, focused on identifying the factors influencing end users as regards self-care management, were professionally transcribed. All the transcribed workshops, and materials such as memos, Post-It notes, and mind maps, were analyzed in accordance with Braun and Clarke’s [42,43] thematic analysis to identify themes or patterns. In this first analysis phase, the data were analyzed through an inductive approach [42]. All data were organized in the NVivo software (QSR International Pvt Ltd) [44], where the thematic analysis was conducted manually in the suggested step-wise fashion [42,43]. The first author (SS) started reading all transcripts, memos, Post-It notes, and mind maps to get familiarized with all the data and gain a better understanding thereof. The memos and summaries were used in the next phase to generate ideas and start to create codes. In searching for themes among the codes, the authors (SS, SB, CF, and ME) first generated subthemes, which were then further consolidated into themes. All subthemes and themes were reviewed and named, going back and forth to the data set. All subthemes and themes were discussed among the authors to reach a consensus in the analysis process.

The data collected from Step 3, including the observation protocols and transcriptions of recorded usability tests, were analyzed and coded inductively in accordance with the thematic analysis process. Then, codes were deductively sorted into a coding matrix that was created using the 4 determinants of the “Use of Technology to Engage in Adaptation by Older Adults and/or Those With Low or Limited Literacy” (USABILITY) framework [45]. A deductive approach was used to gain a more in-depth understanding of the challenges of using video feedback tools at home [36]. The purpose of the USABILITY framework [45] is to assess the prospective usability of health websites among older adults. Here, the framework was used to enhance the understanding of the data collected from the usability tests. The USABILITY framework [45] is grounded in nursing theory, technological theory, and behavioral theory relevant to technology use and usability in the context of older adults. It encompasses four key determinants: (1) learnability, (2) efficiency, (3) perceived user experience, and (4) perceived control.

### Ethical Considerations

This study adhered to the Declaration of Helsinki [46], with all participants getting both verbal and written information about the study design and aim. All participation was voluntary, and the participants could withdraw at any time during the project without giving any explanation. The videos recorded by the end users when using the Move Improve platform at home were deleted and were not used in the study. The study was approved by the Swedish Ethical Review Authority (DNR: 2020-01219).

## Results

### Step 1: Identify End User Needs

**Figure 4:**
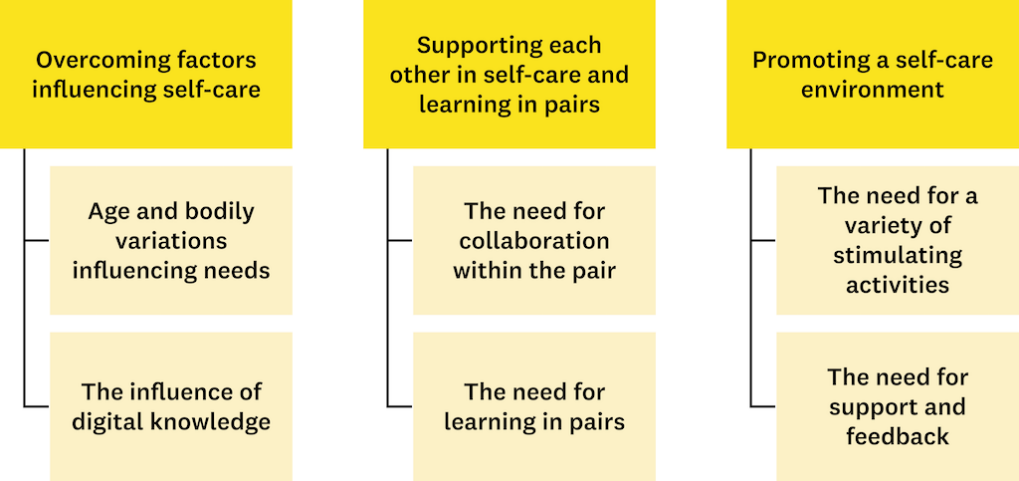
Overview of the 3 themes and 6 subthemes of the thematic analysis of factors influencing self-care management among end users when cocreating a video feedback tool.

The factors influencing self-care management among pairs of older adults were sorted into 3 themes “Overcoming factors influencing self-care,” “Supporting each other in self-care and learning in pairs” and “Promoting a self-care environment,” (Figure 4).

#### Overcoming Factors Influencing Self-Care

The end users expressed a need to overcome factors influencing their self-care management at home. They may encounter diverse challenges to engaging in self-care, including factors such as age and bodily variations that influence their needs. The challenges of self-care activities varied with age and bodily variations, necessitating the recognition and acceptance of limitations. End users mentioned a decline in balance with age, emphasizing a need for proactive balance training. They also mentioned challenges in retrieving items from the floor due to physical impairments and declining balance, hindering their daily lives. The end users described the importance of relaxation, with suggestions of support for breathing exercises emerging during workshops.

I’d like to put it like this, that since I have a heart problem I can’t...I think that I’m exercising and cycling and so on at the edge of my limit. Yeah, that’s what I mean because if I work too hard and my heart can’t keep up and oxygenize my lungs...I used to exercise much more in the past or when I was younger. What I means is that it’s the body itself that’s setting the limit.

End user 5

The end users highlighted another challenge in managing self-care at home: the influence of digital knowledge. Differing levels of familiarity with digital tools were identified among the end users, ranging from daily use of a smartphone to basic use of mobile phones, primarily for calls. They expressed concerns about potential distractions of technology and underlined the challenge of keeping pace with rapid technological advances. Barriers, such as the unreliability of digital tools, including inaccuracies and issues with mobile phones, could lead to difficulties in use, reduced engagement, and a resurgence of previous health issues and symptoms. On the other hand, the end users also expressed an interest in using digital tools to monitor health parameters and self-care activities and thereby improve self-care management. For example, monitoring of step counts and oxygen levels was recognized as having the potential to increase engagement in self-care. However, the end users’ experiences underscored the necessity of better integration of digital tools into daily routines and customizing them based on personal preferences.

#### Supporting Each Other in Self-Care and Learning in Pairs

During the workshops, the end users stated that supporting and learning from each other was a factor influencing self-care management at home. They explained that there was a need for collaboration within the pair, which they thought could be a valuable resource for enhancing self-care management. They also described how they could develop habits to complement each other within a pair over time, based on each person’s unique qualities. This created shared patterns and a degree of flexibility, promoting an environment that valued individual differences and built mutual support within the pair. The end users described a motivating sense of synergy that arose through mutual support, shared experiences, and collaborative activities. This contributed to a sense of enjoyment and underlined the importance of a shared daily routine. In addition, they said that they reminded each other of mutual goals to enhance each other’s motivation when performing different self-care activities at home.

Yeah, but it’s good if you’re two friends, like you are, End user 1 and End user 2. For me ...“Do you want to go for a walk”, “We have to do this now”, that’s like the whip. Because then you can’t let them down either, when you’ve decided “We’re going to do this now.”

End user 6

The end users described a need for learning in pairs, which could increase the motivation or engagement to perform self-care exercises at home. During their use of the structured video feedback at home, they noticed variations in their individual movement patterns, prompting self-reflection on their movements and the self-care exercises. They observed significant differences between their own videos and the demonstration video, underscoring the need for additional practice, particularly in areas such as balance training. The end users highlighted the use of the video feedback tool as an enjoyable shared activity, fostering mutual encouragement in self-care exercises, including mutual learning, and seeking support from one another when facing challenges.

#### Promoting a Self-Care Environment

The end users stated that promoting a self-care environment at home involved recognizing and addressing specific needs. They emphasized the need for a variety of stimulating activities to maintain engagement and motivation in self-care management. According to the end users, a diverse range of stimulating activities was important to help counteract boredom and promote a sustainable practice of self-care when using a video feedback tool. They also recognized the importance of adaptability in the activities, to ensure comfortable performance, and underlined the need for adjusting based on day-to-day status and specific needs. They also stated that the self-care exercises in digital tools needed to contribute to health improvements to ensure use in everyday life.

Yeah, I read beforehand what was included. Balance was one main thing and that’s something that I do struggle with. I use a walker, so I want something that I feel that I can...that it works, so I feel satisfied in that I am able to do these things.

End user 1

Additionally, the end users highlighted the need for support and feedback, viewing it as a factor in enhancing their self-care management. The feedback on self-care exercises when using a video feedback tool as a pair at home could contribute to encouragement and serve as an external reminder. They described a need for support in analyzing strengths and weaknesses when using the video feedback tool, to enhance their understanding of their performance. They thought that adding consistent feedback and follow-up from health care professionals would contribute to effective supervision, with clearly defined goals being essential when managing self-care at home.

### Step 2: Development and Refinement

**Figure 5:**
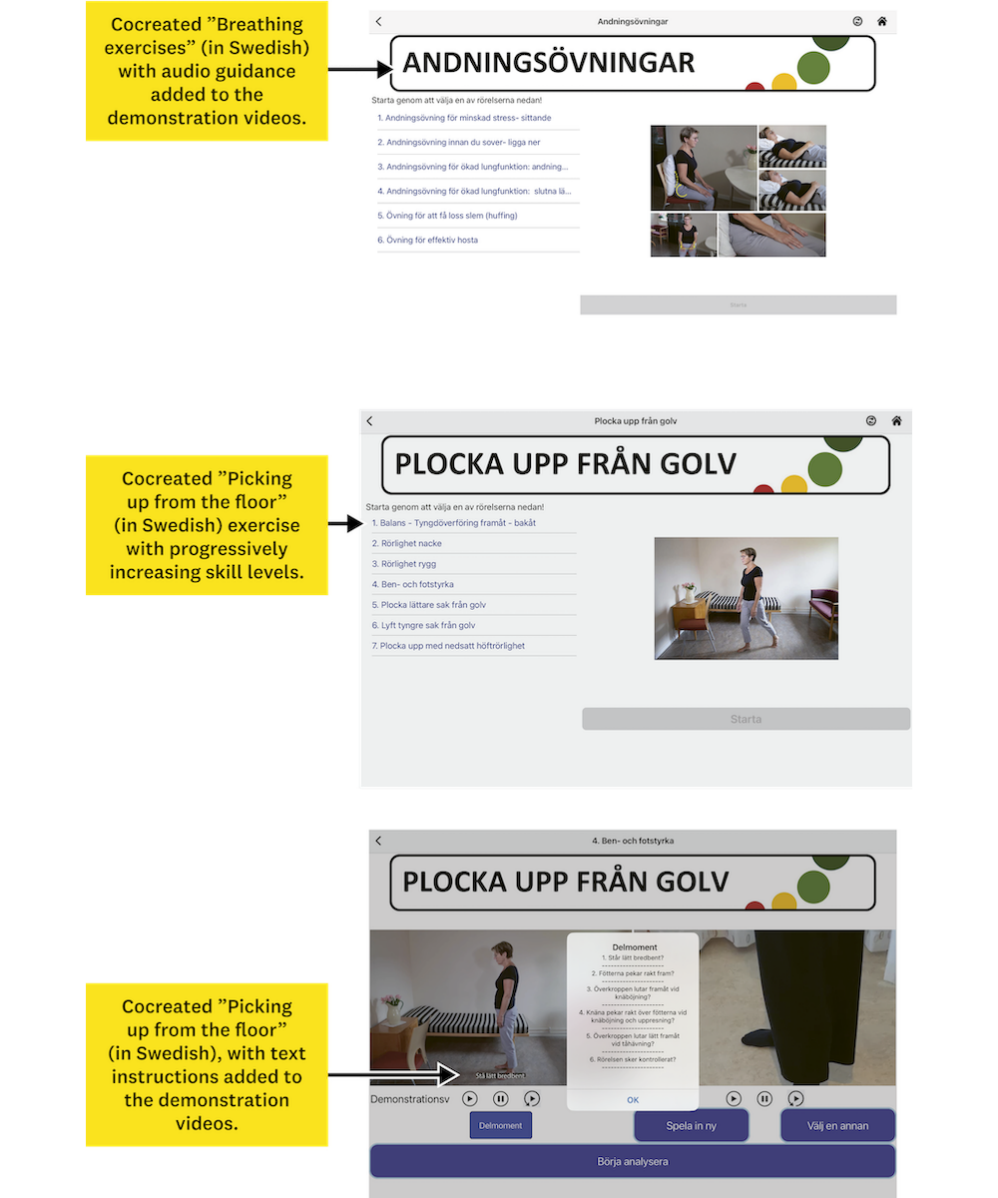
The 2 new cocreated self-care exercises, along with an example of the contents and text instructions for the components in the demonstration videos.

The workshops facilitated the cocreation of new self-care exercises through iterative discussions between the end users and the researchers. The research team decided to develop 2 new self-care exercises in close collaboration with the design team and the end users. These self-care exercises were “Breathing exercises” and “Picking up from the floor,” depicted in Figure 5. The exercise “Picking up from the floor” refers to picking up objects from the floor in a gentle manner. The development and refinement of these exercises aimed to introduce a dynamic and adaptable approach tailored for use by older adults at home. The decision was also made to include audio files in the guide on “Breathing exercises” and text instructions in the video for “Picking up from the floor,” to accommodate hearing or visual impairments. The text instructions gently guided users through each step, which progressively increased in difficulty and range of motion (Figure 5). Manuscripts and instructions for the 2 self-care exercises were created through discussions within the research team and included components specific to each exercise. The research and design teams created and recorded videos and took photographs of the new self-care exercises. These materials were then prepared for use and testing by end users at home. Adjustments and refinements were made by the design team based on end user needs, such as ensuring that instruction videos would not start playing automatically, clarifying instructional images for the different steps, and providing translations from English into Swedish.

### Step 3: Usability Test

Most end users were able to use the video feedback tool, as indicated by the task observation (Table 2). However, some challenges were observed in the recording of self-care exercises. According to the observation, 3 out of 4 participants completed task number 3, which involved recording a video while performing a self-care exercise. One pair of end users needed guidance and hands-on support from the research team to start the recording of the self-care exercise in the video feedback tool during the usability test. The end users often felt uncertain about using the video feedback tool to record exercises performed by their companion, leading to dialogues within end user pairs to collaboratively resolve these issues.

**Table 2:** The task completion rate during the usability tests in Step 3, the end users’ suggestions for improvements, and the research team’s reflection.

Task	Completed task rate	End users’ suggestions for improvements	Research team reflections
1. Start the video feedback tool and choose a self-care exercise	4 of 4 pairs	Larger text and better contrast for visibility. Clear instructions on which exercises are beneficial for specific purposes. Use together with health care professionals to enhance performance and pick correct exercises.	Discussion within the pair about the need for a specific exercise. Difficulties in finding suitable exercises.
2. Prepare reading instructions and watch instruction video	4 of 4 pairs	Hard to understand the instructions, which were described as difficult. Automatic playback of instructional video. Instruction text not visible in the video in portrait mode.	Optimal directions for video recording to capture the exercise performed. Generating interest in doing more self-care exercises.
3. Record the self-care exercise with video	3 of 4 pairs	Need for instruction during the recording of the exercise. A stand for the iPad, to achieve more stable recording during activity execution.	Challenging to fully adapt their movement patterns based on the demonstration video. Difficult to record at home due to difficulty zooming out sufficiently. End users’ fingers partially obstructed the camera, resulting in incomplete video coverage, complicating analysis.
4. Conduct analysis by comparing videos	4 of 4 pairs	Automatic playback of instructional video during analysis.	Struggled to compare their video with the instructional video, which sometimes led to inaccuracies in their analysis responses. End users encountered difficulties in finding their recorded video when beginning the analysis. Easier to use independently on the second attempt.

Some reflections from the research team during the usability test related to language comprehension challenges that arose when the end users encountered error messages in English. The use of the video feedback tool fostered a dialogue within end user pairs, where the end users motivated each other through positive comments or engaged in a friendly competition to achieve the best scores for completed exercises. The end users tended to be less critical of each other and adopted a supportive approach when jointly reviewing the recorded exercise analysis. Some end user pairs performed the analyses of the video recordings together, whereas others completed the analyses individually. During the usability test, the end users provided reflections and insights about their experiences with the video feedback tool, presented in Table 3.

**Table 3:** Summary of usability findings for cocreated self-care exercises, sorted into the coding matrix applying the determinants of the USABILITY (Use of Technology to Engage in Adaptation by Older Adults and/or Those With Low or Limited Literacy) framework.

Element	End users’ reflections
Learnability	Struggled with navigating back within the video feedback tool and returning to the main menu. The demonstration video for the exercise, along with the accompanying images, was considered to clearly illustrate how the exercise should be carried out. The end users liked a combination of text and audio instructions for the exercises.
Efficiency	Confidence in choosing exercise. iPad was heavy and unwieldy during use. It was considered difficult to zoom out sufficiently in recording a video, making it challenging to capture the entire activity when space was a constraint.
Perceived user experience	A need for a digital stylus to enhance precision when interacting with the platform. Challenging to grasp the purpose of the analysis of the recorded exercise. Use involved joint planning and some practicing of the exercise within the pair. Clarified movement patterns and how to improve them.
Perceived control	Uncertainty among the end users on whether they were executing the exercise correctly or understanding it accurately. Instructions for exercise components were unclear (for example, the meaning of “slightly bow-legged” was unclear to the end users). Performing exercises at home eliminated the need to change for gym or health care visits.

### Refinements of the Video Feedback Tool

During the entire iterative EBCD process, minor adjustments and refinements were made in the video feedback tool. One enhancement suggested was related to zoom functionality on the iPad and another was facilitating access to components during analysis.

Examples of adjustments and improvements that were implemented into the platform functionality were as follows: (1) end users need to be able to manually start playback of the video, from both the default interface and specifically during analysis, (2) instructions should be visible in portrait mode, and (3) minor technological errors (bugs) were corrected.

## Discussion

### Principal Findings

Three main findings were identified. First, the results showed that end users can successfully learn and support each other in self-care management and that incorporating video feedback can enhance their motivation and engagement in self-care. By addressing barriers, fostering support and collaboration, and promoting an environment for self-care, adaptive and dynamic self-care exercises were cocreated with end users. Second, we found that there was a discrepancy between the end users and the research team regarding their experiences of choosing self-care exercises in the video feedback tool and understanding their purpose. The end users found it easy to choose exercises, whereas the researchers perceived that they had difficulties with this. The same for the self-care exercises and their purposes; the end users found it challenging to understand the purpose and reason for analyzing exercises, even though the analyses clarified movement differences and training needs. Last, this study showed that it was possible to engage end users as cocreators, whether remotely or in person, for the development of self-care exercises that can be used with video feedback in collaboration with a companion in the comfort of the home.

### Comparison With Previous Work

The video feedback tool in this study was tailored to the needs and preferences of older adults regarding self-care management through the cocreation of 2 new self-care exercises. The perceived user experience was essential for assessing how well the video feedback tool aligned with the needs of the end users [45], as the findings revealed that they understood the variations in their mobility patterns when using the video feedback tool. Moreover, they demonstrated mutual support and engagement during self-care exercises. Similar findings were observed in the study by San et al [30], where increased understanding and engagement in various physical exercises could be noted among children using a video feedback tool. This potential for a sense of collaborative engagement in self-care exercises holds promise for enhancing the overall user experience and for future adoption of this tool.

Taylor et al [47] highlighted that older adults and their companions need to be the focus of attention to reduce the burden of self-care for collaborating pairs. However, in this study, the end users themselves believed that support from health care professionals was needed in selecting self-care exercises and receiving feedback on the exercises performed. Although digital tools hold promises for managing chronic illness among older adults, challenges remain, particularly regarding concerns about their everyday use [47]. Fischer et al [48] emphasized that even if end users are included, this does not necessarily lead to improved adoption of new digital tools within health care services. The use of this video feedback tool in collaboration with health care professionals was not investigated in this study and further studies are needed to enhance integration within health care services.

This study underlined the challenges faced by the end users in choosing and understanding the purpose of self-care exercises in the video feedback tool. This revealed a need for further development and refinements to enhance the efficiency of the tool. The efficiency determinant in the USABILITY framework refers to a system’s ability to enable seamless interactions [45]. This study demonstrated the benefits of using the video feedback tool in pairs, for example by creating a discussion between the end users on which self-care exercises were most appropriate to carry out based on their needs. The mutual support within the pairs enhanced the efficiency in completing tasks using the video feedback tool. Navarro et al [49] suggested that video is an effective method for older adults with chronic illnesses to learn self-care. This finding provides hope for the further implementation of the current video feedback tool in health care services.

This study identified factors influencing self-care management, such as aging and chronic illness, which can alter physical functionality. If such factors compromise end users’ perceived autonomy, confidence, and ability to use a digital tool, they may prevent the use of that tool. Perceived control as described in the USABILITY framework is essential for sustained motivation and use [45]. These findings further underscore the importance of creating digital tools that address the fundamental need for sustained motivation [50], in conjunction with the development of self-care exercises that can promote necessary changes. Cocreating exercises that can motivate older adults to maintain their self-care skills can be the difference between living an independent life and relying on others to live at home [51]. The findings align with other studies suggesting that digital tools should be tailored to older adults’ daily routines and home environments to improve sustainability and long-term usability [7,47,52].

Comparing the results with the USABILITY framework revealed that cocreating digital content with older adults had a positive impact on learnability. This was supported by the end users’ meaningful contributions in refining the content, particularly through suggestions for text and audio instructions related to the 2 new self-care exercises. Learnability also encompasses the end users’ assessments of how easily they could understand the functionalities of the video feedback tool and their experiences of navigating on the platform. Hillis et al [29] showed that peer-to-peer learning through video feedback could facilitate learning and skills acquisition in various physical exercises. However, the results of this study revealed a potential risk that one person in a pair might be overly lenient when analyzing a self-care exercise, which could hinder the other person’s learning process. To mitigate this risk, further research is needed to explore how the video feedback tool can complement existing health care workflows and support structures, as well as be effectively integrated and implemented within health care systems.

### Strengths and Limitations

This study was strengthened by involving end users in all 3 steps of the iterative EBCD process. Incorporating the experiences of older adults and the collective insights of research and design teams from diverse backgrounds broadened the study’s results [33]. Emphasizing early and continuous involvement of older adults in cocreating digital tools aligns with the Medical Research Council’s framework for complex interventions [32]. Additionally, the involvement of multiple researchers analyzing varied data types—such as workshop interviews and usability test observations—strengthened the study’s trustworthiness.

A limitation of this study is the small sample size, which may not fully represent all older adults. In the development of digital tools, it is not feasible to include many participants at once. To address this, a diverse group of older adults, with and without various chronic illnesses and needs, was included (Table 1 presents demographics). Involving older adults with different barriers, such as chronic illness-related impairments, is essential in developing digital tools [10]. A strength of the study is the participation of older adults with both hypertension and heart failure.

Due to COVID-19 restrictions in Sweden, data collection was mainly conducted through remote workshops, which excluded older adults without internet access and created challenges with unreliable internet connections. Limitations included the inability to fully observe verbal communication and body language. Technical issues in one session hindered the participation of one pair of older adults. That session focused primarily on introducing the video feedback tool. Despite these challenges, a key strength of the study was the use of remote data collection via a digital conference system. As noted by Darley and Carroll [5], this facilitates participation from individuals with limited travel options and varied lifestyles.

### Conclusion

Given the growing demand for health care services, the video feedback tool holds the potential to serve as a valuable contribution that can increase patient engagement and motivation in self-care management. This study demonstrated the potential of involving end users as cocreators when developing remote and in-person self-care exercises. The new self-care exercises within the video feedback tool allow older adults to engage with a companion and improve their self-care at home. However, the findings also highlight that older adults need support regarding the use of a video feedback tool, particularly those without a companion to involve or for whom the tool may be challenging to use. Further research is needed to determine how health care professionals can integrate the video feedback tool into their interactions with patients with chronic illness and their companions. Future studies should investigate the application and adaptation of video feedback in health care settings, focusing on collaboration between health care professionals and older adults to enhance self-care at home.

## Appendix

Multimedia Appendix 1 Demonstration of Move Improve, where self-assessment and peer assessment are conducted with guidance from key component questions, with the overarching goal of mastering a skill.

## Abbreviations

EBCD: experience-based co-design
USABILITY: Use of Technology to Engage in Adaptation by Older Adults and/or Those With Low or Limited Literacy

## Data Availability

The data that were collected and analyzed in this manuscript are not publicly available due to participants not having consented to public availability. Aggregated data in Swedish are available from the corresponding author on reasonable request.
